# *TP53* Mutations Identified Using NGS Comprise the Overwhelming Majority of *TP53* Disruptions in CLL: Results From a Multicentre Study

**DOI:** 10.3389/fonc.2022.909615

**Published:** 2022-06-28

**Authors:** Mark A. Catherwood, Dorte Wren, Laura Chiecchio, Doriane Cavalieri, David Donaldson, Sarah Lawless, Ezzat ElHassadi, Amjad Hayat, Mary R. Cahill, Derville O’Shea, Jeremy Sargent, Peter Stewart, Manisha Maurya, John Quinn, Philip Murphy, David Gonzalez de Castro, Ken Mills, Nicholas C. P. Cross, Francesco Forconi, Sunil Iyengar, Anna Schuh, Patrick Thornton

**Affiliations:** ^1^ Haematology Department, Belfast Health and Social Care Trust, Belfast, United Kingdom; ^2^ The Royal Marsden Hospital and the Institute of Cancer Research, Biomedical Research Centre, London, United Kingdom; ^3^ Wessex Regional Genetics Laboratory, Salisbury National Health Service (NHS) Foundation Trust, Salisbury, United Kingdom; ^4^ Oxford Molecular Diagnostics Centre, Oxford University Hospitals, Oxford, United Kingdom; ^5^ Department of Haematology, University Hospital Waterford, Waterford, Ireland; ^6^ Department of Haematology, University Hospital Galway, Galway, Ireland; ^7^ Department of Haematology, Cork University Hospital, Cork, Ireland; ^8^ Department of Haematology, Our Lady of Lourdes Hospital, Queens University Belfast, Drogheda, Ireland; ^9^ Centre for Cancer Research and Cell Biology (CCRCB), Queen’s University Belfast, Belfast, United Kingdom; ^10^ Department of Haematology, Beaumont Hospital, Dublin, Ireland; ^11^ Faculty of Medicine, University of Southampton, Southampton, United Kingdom

**Keywords:** chronic lymphocytic leukaemia, p53, deletion 17p, prognosis, next generation sequencing

## Abstract

Limited data exists to show the correlation of (tumour protein 53) *TP53* mutation detected by Next generation sequencing (NGS) and the presence/absence of deletions of 17p13 detected by FISH. The study which is the largest series to date includes 2332 CLL patients referred for analysis of del(17p) by FISH and *TP53* mutations by NGS before treatment. Using a 10% variant allele frequency (VAF) threshold, cases were segregated into high burden mutations (≥10%) and low burden mutations (<10%). *TP53* aberrations (17p [del(17p)] and/or *TP53* mutation) were detected in 320/2332 patients (13.7%). Using NGS analysis, 429 *TP53* mutations were identified in 303 patients (13%). Of these 238 (79%) and 65 (21%) were cases with high burden and low burden mutations respectively. In our cohort, 2012 cases did not demonstrate a *TP53* aberration (86.3%). A total of 159 cases showed *TP53* mutations in the absence of del(17p) (49/159 with low burden *TP53* mutations) and 144 cases had both *TP53* mutation and del(17p) (16/144 with low burden mutations). Only 17/2332 (0.7%) cases demonstrated del(17p) with no *TP53* mutation. Validated NGS protocols should be used in clinical decision making to avoid missing low-burden *TP53* mutations and can detect the vast majority of *TP53* aberrations.

## Introduction

Deletion of chromosome 17p [del(17p)] and *TP53* mutation (*TP53* mut) referred to as *TP53* aberrations can be found in 8%–10% of previously untreated chronic lymphocytic leukaemia (CLL) patients and in up to 30%–40% of relapsed/refractory cases. *TP53* aberrations represent the most relevant risk factors for both progression free and overall survival following chemoimmunotherapy (1, 2). The introduction of small molecule inhibitors has led to enhanced response rates in patients with *TP53* aberrations (3–5). Therefore, the identification of *TP53* aberrations is essential for determining treatment decisions in CLL (6, 7). Historical data using Sanger sequencing suggests that approximately 80% of patients with del(17p) also carry a mutation in the second allele (8). A subset of patients also exhibits *TP53* mut without del(17p) (8).

The assessment of del(17p) is routinely performed by Fluorescence *in situ* hybridization (FISH). The cut-off for a positive result varies within laboratories with the threshold >20% of cells with del(17p) deemed to be a clinically relevant clone (9). However, it is recognized that a subset of patients with del(17p) have stable disease without the need for treatment (10).

Sanger sequencing is widely used for *TP53* mutational analysis, however it may misclassify cases of *TP53* mutations as wildtype when variants with allelic frequencies below the detection limit of Sanger sequencing are present. Recent studies using next generation sequencing (NGS) have shown that *TP53* mutations can be present at low clonal abundance in tumour cell populations, termed low-burden and have in certain studies the same detrimental effect on disease course (11–13). Therefore updated guidelines from the *TP53* network of ERIC (European Research Initiative on CLL- www.ericcll.org) suggest a threshold of 10% allelic burden for reporting mutations detected by NGS segregating these into high burden (≥10 variant allele frequency (VAF)) and low burden (<10% VAF) mutation (6). In the literature, contradictory results exist regarding the biological relevance of low burden mutations in CLL. This in part may be due to various sequencing strategies.

Therefore, the aim of this study, which is the largest cohort to date, was to investigate the presence of low and high burden *TP53* mutations in a “real-world” cohort of 2332 CLL cases using sensitive NGS and to correlate results with FISH data.

## Methods

Pretreatment peripheral blood samples from 2332 CLL patients referred for analysis of del(17p13) by FISH and *TP53* mutations by NGS were available for the present study diagnosed between 2015-2019. A retrospective audit of *TP53* status was undertaken. Best practice in the UK follows established guidelines, meaning that *TP53* testing is recommended prior to each line of treatment but not at diagnosis. As participants are part of Specialized HaemOnc Diagnostics services, requests for *TP53* testing in newly diagnosed patients would automatically be rejected.

The study was conducted according to the Declaration of Helsinki. Patients were diagnosed according to iwCLL guidelines (14). In all cases, analysis was performed on DNA obtained from >50% tumour cells. FISH analysis for del(17p13) was performed using Vysis Probes with a 10% cut-off for a positive result. *TP53* mutation screening was performed by NGS with a panel covering exons 2-11 as previously described (15) or by an Illumina amplicon-based strategy. Briefly, the amplicon-based panel was a bespoke assay and amplicon libraries for are generated by Reverse Complement PCR (RC-PCR) technology. The technique permits both the amplification and the ability to append sequences or functional domains of choice independently to either end of the generated amplicons in a single closed tube reaction. Primers for the *TP53* assay were designed in house and sequencing was performed on the Miseq using Illumina chemistry. Raw data was aligned using GATK. Indels are realigned using GeminiMulti indel realigner and Pisces is used for variant calling (both Illumina). Normally analyses with read depths below 5000 are failed.

A VAF cut-off of 1% was used to exclude false positive variants within the cohorts. Pathogenicity assessment of all variants was performed according to ERIC guidelines (6).

## Results

**Figure 1:**
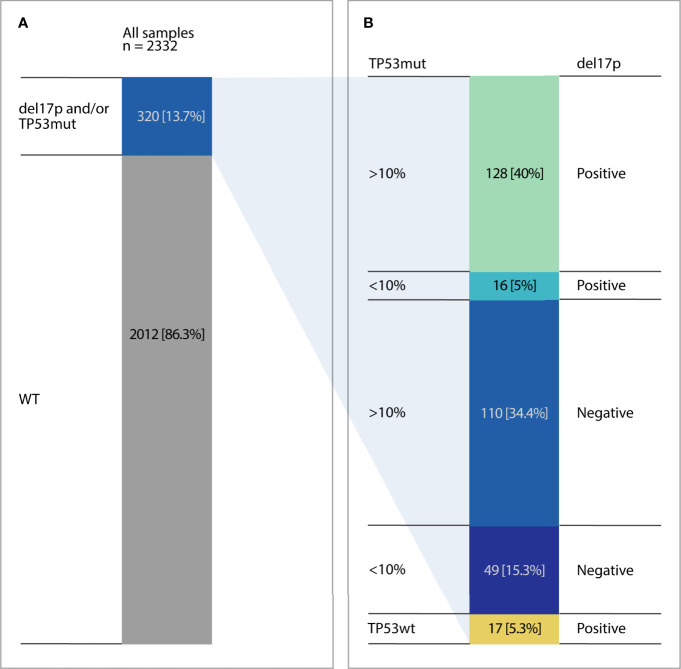
*TP53* aberrations in the analyzed cohort. Composition of *TP53* defects.

**Figure 2:**
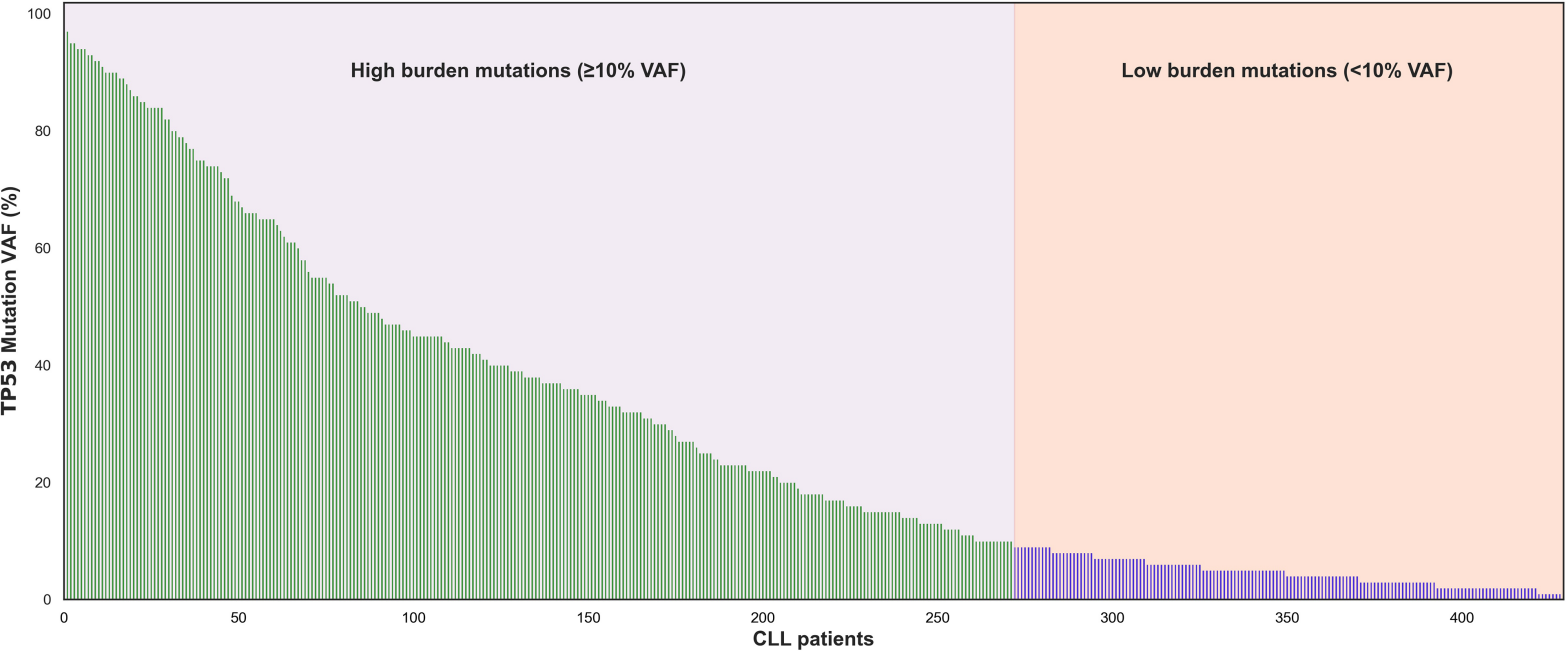
Molecular Profile of *TP53* mutations in the cohort. Using a cutoff of 10% VAF 271 *TP53* mutations (228 patients) had high burden mutations and 158 *TP53* mutations (65 patients) had low burden mutations.

Altogether 2332 patients entering first line treatment were included in this study with *TP53* aberrations detected in 320/2332 patients (**Figures 1A, B**). Using NGS analysis, 429 *TP53* mutations were identified in 303 patients (13%). More than one *TP53* mutation was detected in 76 patients (2-8 mutations per patient, **Supplementary Table 1**). When considering all 429 *TP53* mutations in the cohort the VAF ranged from 1-97%; mean 28%. Using the 10% VAF threshold, cases were segregated into high burden mutations (≥10%) and low burden mutations (<10%). The high and low burden separation was based on the VAF of the most prevalent *TP53* mutation. 271 (63%) were classified as high burden mutations (VAF range: 10-97%; mean 42%). 158 were classified as low burden mutations (VAF range: 1-9%; mean 5%) (**Figure 2**).

**Figure 3:**
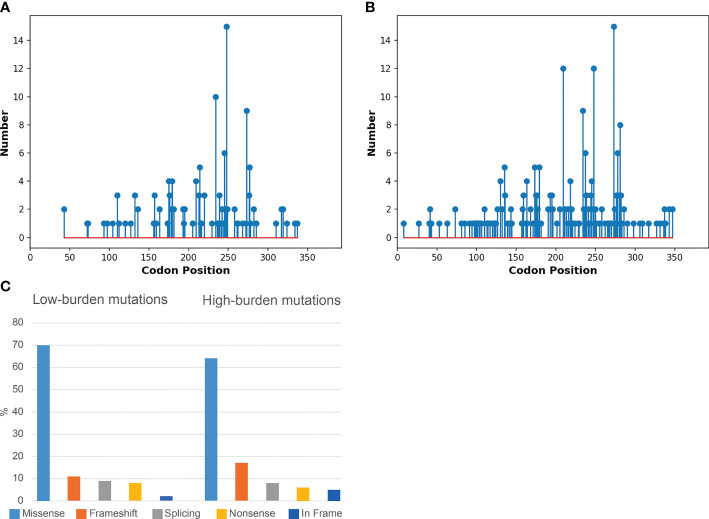
Molecular Profile of *TP53* mutations in low and high burden cohorts. **(A)** Needle plot graph of low burden *TP53* mutations along the *TP53* coding sequence. **(B)** Needle plot graph of high burden *TP53* mutations along the *TP53* coding sequence. **(C)** Bar chart of mutations effect on the p53 protein in terms of amino acid changes in the low and high burden context.

This translated into 238 patients classified as high burden cases and 65 identified as low burden cases (**Supplementary Table 1**). The needle plot graphs demonstrated no differences in *TP53* coding mutations between high and low burden cases (**Figures 3A, B**). The mutation profile revealed that the majority of mutations were missense mutations followed by frameshift, splicing and nonsense mutations and is in keeping with previous reports (**Figure 3C**) (16, 17). No significant difference within mutation type existed between the low and high burden groups (P=0.5). The amino acid most frequently mutated were at positions 175, 209, 234, 248 and 273 indicating the classical hot spot mutations in CLL. Codons 175, 209, 234, 248 and 273 represented 110/429 (25%) mutations in the total cohort and showed similar allocation in low and high burden case (**Figures 3A, B**).

Combining FISH data on del(17p) with *TP53* mutation data in our cohort, 2012 cases did not demonstrate a *TP53* aberration (86.3%). However, 17 cases demonstrated del(17p) only (0.7%). Average del(17p) was 40% (range 10-91%) in del(17p) only cases and was significantly higher in del(17p)/*TP53* mut cases (55% (range 10-100%: p<0.05). One hundred and fifty-nine patients (159) were *TP53* mutated only cases (49/159 with low burden TP53 mutations) and 144 cases with both del(17p) and *TP53* mutation (16/144 with low burden mutations, **Figure 1B**).

## Discussion

In this study, which is the largest study to date assessing *TP53* aberrations for both del(17p) and *TP53* mutation by NGS in cases of treatment naïve CLL. Using NGS analysis, 429 *TP53* mutations were identified in 303 patients (13%). Current guidelines from the *TP53* network of ERIC suggest a threshold of 10% allelic burden for reporting mutations detected by NGS (6). An acknowledgement is made in reference to cases with 5-10% VAF. In this study we employed a threshold of 10% VAF separating the cohort into high and low burden subgroups. High burden mutations were evident in 10.2% (238 cases) and low burden mutations in 2.8% (65 cases). This figure is lower than that reported in other studies and is likely due to the threshold of 1% used in this study (12, 13). Even with this threshold, 49 cases [*TP53* mut/del(17p) wt] in this cohort would have been misclassified as *TP53* proficient cases. This is an important observation given the recent publication that clearly demonstrates a shorter survival in cases with VAFs of 5-10% (13). This study again questions the threshold of 10% VAF and the impact this has in the misclassification of *TP53* aberrations.

Whilst most tumour suppressors are inactivated by frameshift or nonsense mutations, the most frequent mode of inactivation of *TP53* in CLL is by missense mutations which is a unique phenomenon. The mutation profile of the cohort did not differ when separated into high and low burden mutations. The vast majority of mutations were missense and no significant differences were observed between the low and high burden cohorts (**Figures 3A–C**). Unique to CLL is the presence of a specific hot spot variant leading to premature termination [p.(R209Kfs*6)]. This specific variant was demonstrated both in low and high burden cases highlighting the similar mutation profile between the cohorts (**Figures 3A, B**). The majority of *TP53* mutations are located within the DNA binding domain of the gene and hot spot mutations are frequently observed in CLL. This study showed an enrichment of mutations in codons 175, 209, 234, 248 and 273 representing (25%) of all mutations in the total cohort. A similar pattern was evident in both low and high burden subgroups confirming the disease specific *TP53* mutation profile in CLL (**Figures 3A, B**) (16).

Combining FISH data on del(17p) with *TP53* mutation data in our cohort, 2012 cases did not demonstrate a *TP53* aberration (86.3%) whereas *TP53* aberrations were detected in 13.7% of patients. This is in keeping with recent data from independent groups that utilized various NGS strategies and bioinformatics pipelines (11, 13, 18).

In this study we have demonstrated the existence of del(17p) in the absence of a *TP53* mutation in 17/2332 (0.7%) which is in keeping with the literature (1, 13). The average del(17p) clone was 40% with a range of 10-91% (**Supplementary Table 1**) with 8/17 cases having a del(17p) clone less than 25%. Patients in population based cohorts are still routinely screened for del(17p) by FISH, whilst testing for *TP53* mutations can vary substantially by institution. This is despite very clear guidelines to the contrary (6, 14). Screening for only del(17p) in our study would have missed 50% of the alterations in the cohort (159/320). The relevance of FISH only based studies in the era of NGS is questionable as only a minority of p53 deficient cases are missed by NGS. In this series 0.7% of p53 deficient cases were missed by NGS of which 8 cases had a del(17p) clone size of less than 25%. Also recent data showing low-frequency del(17p) sub clones (<25% of CLL cells) in the absence of a *TP53* mutation has been demonstrated to mirror that of cases with no del(17p) in the chemoimmunotherapy setting (12, 19). In the study by Do et al. 15/20 (75%) patients demonstrated a low frequency subclone of del(17p) (<25)). This is a well recognized phenomena in the literature with subset of patients with low frequency del(17p) clones having enhanced progression free survival (10). This subgroup of patients is enriched with a mutated IGHV gene and relatively few copy number alterations. The study by Do et al. represents a surprisingly high percentage of low level del(17p) not previously described and likely reflects the genomic composition of the elderly trial cohort in the study. In the current study, we demonstrated 28/144 (19%) cases where del(17p) <25% with 17 cases demonstrating a high burden (≥10%) mutations and 11 cases with low burden mutations (**Figure 1B**). Unfortunately clinical data was not available in this study to ascertain the IGHV status in the cohort of del(17p) subclones.

*TP53* aberrations are still relevant in the era of novel therapies. Long term survival outcomes remain inferior in cohorts of patients with *TP53* aberrations (20, 21). This is likely attributable to the role of p53 in the maintenance of genomic stability. It is well recognized that mutations in *TP53* occur early in the disease progression proceeding the genomic instability generated by chromosomal abnormalities.

This has been further addresses in a recent study demonstrating that patients treated with single-agent ibrutinib carrying only a single *TP53* hit have a superior long term response while multi-hit *TP53* is associated with a shorter progression free and overall survival (22). In this scenario single hit CLL can be classified by the presence of either del(17p) or *TP53* mut. Multi hit CLL arises when either del(17p) and *TP53* mut occur together or when greater than one *TP53* mutation is found. Whilst this is of interest it has yet to be verified in larger cohorts or indeed in separate treatment regimens. In our current study 55% (176/320) were single hit with 45% (144/320) of cases demonstrating a multi hit CLL. In this study ≥2 *TP53* mutations were detected in 76 patients with the majority of cases in the *TP53* mut/del(17p) wt cohort (46/76) with the remaining 30 cases in the *TP53* mut/no del(17p) cohort. This reinforces the need to redefine a VAF threshold to aid in the selection of *TP53* mutated patients benefiting from targeted treatments.

In conclusion, in the largest series to date we have demonstrated the presence of low and high burden *TP53* mutations in a series of CLL cases. The use of NGS prevents cases being misclassified as normal TP53 due to its enhanced sensitivity. In the investigation of *TP53* aberrations, NGS is an important strategy for patient management in this setting.

## Data Availability Statement

The original contributions presented in the study are included in the article/**Supplementary Material**, further inquiries can be directed to the corresponding author/s.

## Ethics Statement

Using the NHS Health Research Authority decision tools this study classes as clinical audit (i.e., no randomization of patients, no alteration to standard clinical care and informs practice in our setting) and therefore formal research ethics is not required.

## Author Contributions

MAC, AS and PT conceived the project. DW, LC, DC, PS, MM and DC performed data analysis. DD, SL, EE, AH, MRC, DO’S, JQ, PM, JS, KM, FF, SI, NC, AS and PT provided clinical input. All authors read and agreed with the manuscript.

## Funding

FF work is funded by the Cancer Research UK ECRIN-M3 accelerator award C42023/A29370.

## Conflict of Interest

Author SL has received honoraria from Abbvie, Janssen, BMS/Celegene and Sanofi. DG has received honoraria, consultancy and/or research funding from Roche, AstraZeneca, Novartis, Janssen, Elli Lilly, Incyte, Promega and Illumina and is founder of Univ8 Genomics Ltd. Author NC has received honoraria from Novartis, Incyte and Astellas and research support from Novartis. Author AS received honoraria from Astra Zeneca, Janssen, Roche, Adaptive Biotechnology, Exact Sciences and AbbVie, and received nonrestricted educational grants from Astra Zeneca and Janssen and in-kind contributions from Illumina and Oxford Nanopore technologies. Author FF received honoraria from Abbvie, Janssen-cilag, Beigene, Astra-Zeneca and BC platform.

The remaining authors declare that the research was conducted in the absence of any commercial or financial relationships that could be construed as a potential conflict of interest.

## Publisher’s Note

All claims expressed in this article are solely those of the authors and do not necessarily represent those of their affiliated organizations, or those of the publisher, the editors and the reviewers. Any product that may be evaluated in this article, or claim that may be made by its manufacturer, is not guaranteed or endorsed by the publisher.

## Supplementary Material

Supplementary Table 1
*TP53* mutations from mutated cohort.

Supplementary Table 2 Sequencing metrics from Amplicon and Capture technologies.
